# Supplementary material for: Transitions and challenges for people with Parkinson’s and their family members: A qualitative study
Source: PLoS One. 2022 Jul 18;17(7):e0268588. doi: 10.1371/journal.pone.0268588 (PMC9292070; doi:10.1371/journal.pone.0268588)
Supplement: S1 File — (DOCX) [file pone.0268588.s002.docx]

**Topic guides**

**TOPIC GUIDE 1.1: Patients:**

1. **[Current condition and self-management of Parkinson’s on a daily basis; what are the difficulties?]**

- **Can you tell me what a normal day looks like for you?**
- **How do you feel that you are managing your Parkinson’s?**
  - Which are the most difficult aspects of your Parkinson’s for you at the moment?
  - If you are finding your Parkinson’s difficult what do you do?
  - Recap: You’ve spoken about x,y z. Sometimes people with Parkinson’s have difficulty with some of the following:
- Exercise,
- Unsteadiness on their feet and falling,
- Tiredness (falling asleep during the day, lack of energy limiting things you’d like to do),
- Changes in their mood (lost interest in the world around you or things you used to enjoy? Feel more anxious than usual? Have lower moods, or flat moods)
- Seeing things that are not there? Or have beliefs that they know not to be true?(*hallucinations)*,
- Problems remembering things, or keeping concentration, forget to do things
- Intimate relationships can change
- Finding it difficult to swallow food,
- Changes in toileting (passing water more regularly/ at night; constipation)
- Not being able to taste or smell so well
- Experiences of pain
- Find you sweat more
- If you have experienced any of these what do you do ?
- What helps you with that/ What resouces have helped? (pos/neg/ fears/concerns)
  - Are there ways of coping that you've used previously that you can no longer use now?

Ie., diet, exercise, mobility, social, mood (how relevant are these areas to your situation?)

1. **[****Current help in the management of Parkinson’s.]**

- **Is managing your Parkinson’s something you ever talk about with others?**
- If so, who?
- What do you discuss?
- **What healthcare professionals and services are available that are of most help to manage your Parkinson’s at home?**
- How do you access services (ie., when things are difficult who to you go to?)
- How do you feel about accessing services? (easy, difficult to access?)
- Where would you prefer this type of service to be based?
- What would help you stay as independent at home?
- **What do you think, if anything, gets in the way of you managing your condition as well as possible?**
- (ie, time, what, who, how could that be overcome)
- **What are your views about the help and information, resources that is available?**
- How do you find (get hold of) information and advice?
- What do you find most helpful/ least helpful?
- What makes it the most/least helpful?

(Following up on issues raised earlier) How have you found the help and information that has been available at different times of having Parkinson’s?:

- At diagnosis – who you tell, how, support of children/ carers
- Early stages
- Comorbidities

1. **Hospital Admissions**

- **Have there been times when your Parkinson’s proved difficult and you had to go in to hospital?**

If applicable:

- **Can you tell me about it/the last time you were admitted to hospital?**

ie., Reason for admission, length of admission, who called for help etc

- - Can you tell me about your experience during hospital?
  - What went well? Was there anything that could be improved in relation to your PD?
- **Looking back, was there anything that could have been done to prevent it/avoid going in to hospital**?
- **What happened when you were discharged**?
  - What kind of help did you get for the problem at home?
  - What plans were put in place?
- **Is there anything more generally you think could be done to help prevent people with PD being admitted to hospital?**

**Any other comments?**

**Thank you!**

**TOPIC GUIDE 1.2: Carers:**

1. **[Current situation]**

- **Can you tell me about your role in relation to the person you care for**
- who you are caring for
- how they are
- what the difficulties and challenges are

Sometimes people with Parkinson’s have difficulty with some of the following, is that the case for the person you care for?

- Exercise,
- Unsteadiness on their feet and falling,
- Tiredness (falling asleep during the day, lack of energy limiting things you’d like to do),
- Changes in their mood (lost interest in the world around you or things you used to enjoy? Feel more anxious than usual? Have lower moods, or flat moods)
- Seeing things that are not there? Or have beliefs that they know not to be true?(*hallucinations)*,
- Problems remembering things, or keeping concentration, forget to do things
- Intimate relationships can change
- Finding it difficult to swallow food,
- Changes in toileting (passing water more regularly/ at night; constipation)
- Not being able to taste or smell so well
- Experiences of pain
- Find you sweat more

If they have experienced any of these what do you do ?

What helps them, and you with that/ What resouces have helped? (pos/neg/ fears/concerns)

- **What does the person you are supporting currently do to stay healthy and manage their Parkinson’s?**

ie., diet, exercise, mobility, social, mood, self management (how relevant are these areas to your situation?)

1. **[Views on maintaining well-being and independence]**

- **What might help** [person caring for**] continue to do what they want to do/enjoy doing for as long as possible?**
- How can [they] be best supported to remain independent/retain their current level of functioning and independence [as applicable]?
- How can friends and family help people with Parkinson’s achieve this?
- What do you think can help or encourage people with Parkinson’s to make changes to become healthier? – give any examples?
- **What do you think, if anything, gets in the way of people with Parkinson’s managing their condition as well as possible?**
- What could be done to overcome these hinderances?

1. **[Views on what help/ information/resources are available**]

- **What is your experience of any existing advice, information, and self management resources for managing Parkinson’s?**

ie., What information/resources have been used

- **What are your views about the help, information, resources that are available?**
- How do you find (get hold of) information and advice?
- What do you find most helpful/ least helpful?
- What makes it the most/least helpful?
- **What sorts of things do you need help with, and information about?**
- What advice have you received?

(Following up on issues raised earlier) How have you found the help and information that has been available at different times of having Parkinson’s?:

- At diagnosis – who you tell, how, support of children/ carers
- Early stages
- Comorbidities
- **Is managing the Parkinson’s something you ever talk about with others?**
- If so, who?
- What do you discuss?
- What sorts of things do you need help with?
- **What healthcare professionals and services are available that are of most help to manage your Parkinson’s at home?**
- How do you access services (ie., when things are difficult who to you go to?)
- How do you feel about accessing services? (easy, difficult to access?)

1. **Hospital Admissions]**

**Have there been times when the person you are supporting has had to go in to hospital?**

If applicable:

- **Can you tell me about it/the last time the person you are caring for was admitted to hospital?**
- Reason for admission, length of admission, who called for help etc?
  - What went well? Was there anything that could be improved in relation to to the PD of the person you support?
- **Looking back, was there anything that could have been done to prevent /avoid going in to hospital?**
- could have been dealt with better with them staying at home (eg with more help at home from GP, nurse etc)? If yes, can you tell me more about what happened?
- **What happened when they were discharged?**
  - What kind of help did you get for the problem at home?
  - What plans were put in place?
- **Is there anything more generally you think should be done to prevent people with Parkinson’s being admitted to hospital?**

**Any other comments?**

**Thank you!**
